# Metagenomic Analysis of Bacterial Communities in Agricultural Soils from Vietnam with Special Attention to Phosphate Solubilizing Bacteria

**DOI:** 10.3390/microorganisms9091796

**Published:** 2021-08-24

**Authors:** Anna Hegyi, Tran Bao Khuyen Nguyen, Katalin Posta

**Affiliations:** Department of Microbiology and Applied Biotechnology, Institute of Genetics and Biotechnology, Hungarian University of Agriculture and Life Sciences, 2100 Gödöllő, Hungary; hegyian@gmail.com (A.H.); ntbkhuyen@hcmunre.edu.vn (T.B.K.N.)

**Keywords:** phosphate solubilizing bacteria, *phoD* gene, alkaline phosphatase activity, next generation sequencing

## Abstract

Bacterial communities can promote increased phosphorus (P) availability for plants and microbes in soil via various mechanisms of phosphate solubilization. The production of extracellular phosphatases releases available P through the hydrolysis of organic P. Examining the abundance and diversity of the bacterial community, including phosphate solubilizing bacteria in soil, may provide valuable information to overcome P scarcity in soil ecosystems. Here, the diversity and relative abundance of bacterial phyla and genera of six agricultural soil samples from Vietnam were analysed by next generation sequencing of the 16S rRNA gene. Phosphatase activities of each soil were compared with physico-chemical parameters and the abundance of the alkaline phosphatase gene *phoD*. We showed the dominance of Chloroflexi, Proteobacteria, Actinobacteria, Acidobacteria and Firmicutes. Total nitrogen positively correlated with phyla Proteobacteria, Acidobacteria, Firmicutes and Planctomycetes. The abundance of several genera of Proteobacteria showed positive relationship with the copy number of the *phoD* gene. The abundance of several taxa positively correlated with silt content, while a negative relationship of Proteobacteria was found with sand content. Our results demonstrated the clear influence of soil physico-chemical properties on the abundance of various bacterial taxa including those potentially involved in phosphate solubilization.

## 1. Introduction

Phosphorus (P) is an indispensable element for nucleic acids, nucleotides, phospholipids and enzymes. Thus, it is essential for the biochemical and physiological processes of all forms of life. After nitrogen, it is the second most important macronutrient for plants. The phosphorus content in soil originates from the chemical weathering of phosphorus-bearing rocks, atmospheric deposition and plant residue [1,2]. However, the main sources of recoverable P are not renewable, which are predicted to run out in 80 years [3]. Besides, only 0.1% of soil P content is available for plants as only dissolved phosphate can be assimilated by soil biota [4]. Therefore, most terrestrial ecosystems have to deal with P scarcity. In order to overcome the negative impacts of P deficiency on agricultural productivity and to increase crop yields, conventional farming systems use P fertilizers. However, often only 10–25% of applied P fertilizers are available for plants [5], while the remaining amount of P may lead to environmental issues, such as waterway eutrophication [6]. Therefore, an investigation of new strategies in agriculture is ongoing to improve the efficiency of phosphorus utilization entailing higher crop yields as well as reduced environmental pollution.

Microbial P solubilization and mineralization could be an alternative way to release bioavailable phosphorus from total soil phosphorus by using phosphate solubilizing microbes (PSM) as biofertilizers [7,8]. PSMs are able to solubilize inorganic phosphorus and mineralize insoluble organic phosphorus. The mineralization ability of numerous soil microorganisms has been reported including bacteria, fungi, actinomycetes and algae. Solubilization mainly occurs by the microbial production of organic acids, while the principal mechanism of mineralization is the production of extracellular phosphatases [7].

Alkaline (AlP, EC 3.1.3.1) and acid (AcP, EC 3.1.3.2) phosphatases hydrolyse inorganic orthophosphate ions from organic phosphate, which are readily available for plants and microorganisms from soil solution. Soil phosphatase activity is influenced by several factors, such as the temperature, moisture, nitrogen content of soil, as well as P demand of the biota and P availability. In addition to microorganisms, phosphatase enzymes can be produced by plant roots in soil [9]. Genes encoding alkaline phosphatase are mainly found in bacterial taxa such as Actinobacteria, Gemmatimonadetes, Spirochaetes and Verrucomicrobia [10]. Most of these microbes carry the phosphatase (Pho) regulon containing coregulated genes involved in the synthesis of phosphatases, phosphate transporters and phosphonate utilization. Bacterial AlP production was reported to be coded as part of the Pho regulon by one of three homologue gene families (*phoA*, *phoD*, *phoX*) [11,12,13,14]. Metagenomic studies revealed that 32% of sequenced prokaryotic genomes contain at least one of the *phoA*, *phoD* or *phoX* genes, among which *phoD* is the most abundant bacterial AlP gene [15]. P starvation was shown to induce certain components of Pho regulon, which enabled the use of inorganic P and alternative P sources [16]. The addition of certain PSM was shown to increase the abundances of organic and inorganic P cycling-related genes, including *phoD* [17].

Investigation of the abundance of the bacterial *phoD* gene in soil revealed positive correlation of the *phoD* copy number with soil alkaline phosphatase activity, but other parameters were also shown to affect it, such as soil management or labile P content [18,19,20]. Analysis of the *phoD* bacterial phosphatase gene, soil alkaline phosphatase activities and available P content may help to better understand the biological P transformation mechanism, P availability and plant uptake.

Countries whose agriculture accounts for a considerable part of GDP are exposed the most to the danger of limited P availability in soil. Vietnam is among these countries and is also highly exposed to the negative effects of climate change and soil erosion [21]. The proportion of agricultural area has increased in several areas, such as in Quang Tri province, in the period from 2015 to 2019 [22]. Besides, intensive inorganic fertilizer use and plant protection products have led to soil degradation, heavy metal accumulation, increased soil acidity and the accumulation of organochlorine pesticides. The increased use of plant protection products and their potential accumulation in agricultural products, soil and water represent an environmental risk [23]. Additionally, plant protection chemicals can have a negative impact on soil microbial functions and biochemical processes [24]. Only a few studies have investigated the microbial diversity of Vietnamese agricultural soil, which have mainly focused on the rice paddy fields of the Mekong delta [25,26]. In addition, we have no information about the metagenomic analysis of bacterial communities from Vietnamese soil samples using next generation sequencing. Thus, it would be of interest to learn more about the microbial communities of agricultural soil from the northern parts of Vietnam. Investigation of microbial communities and an understanding of P solubilization/mineralization mechanisms in soils in Vietnam might help to overcome P scarcity and to develop better agricultural practices, including the use of PSM biofertilizers in the future.

In this study, we analysed the diversity and relative abundance of the bacterial taxa of six soil samples from Quang Tri province (Vietnam) with special attention to potential phosphate solubilizing bacteria. We compared the phosphatase activities of soil samples with various physico-chemical parameters and the abundance of the *phoD* copy number.

## 2. Materials and Methods

### 2.1. Site Description, Soil Sampling and Analysis

Soil samples were collected at six locations across Quang Tri province in Vinh Linh and Cam Lo town, Vietnam in August 2019 (rainy season). Sampling sites were plantations growing various annual and perennial crops (Table S1). The area has a tropical monsoon climate, an annual average temperature of 24–25 °C and an annual average precipitation of 2200–2500 mm (“Vietnam Association for Safe Water and Environmental Sanitation” 2015).

Soil samples (n = 6, 0–40 cm) with five replicates were collected using a regular soil auger (5.715 cm diameter). Five subsamples of identical soil were homogenized by sieving (4 mm), mixed to form a composite sample and then sealed in sterile plastic bags. A portion of each sample was sent to the Center for Monitoring Natural Resources and Environment (Quang Tri, Vietnam) for laboratory analysis. Soil pH was measured by a glass pH electrode in a soil-to-KCl (1:5 *w*/*v*) mixture of dry soil and KCl 1 mol/L solution. Soil organic carbon (SOC) was determined via oxidation by potassium dichromate in sulfuric acid solution followed by Fe^2+^ titration (Walkley–Black method) [27]. Total nitrogen was measured using Kjeldahl digestion with a substitution for selenium with titanium dioxide [28]. Total phosphorus was measured by spectrophotometric analysis after digestion with sulfuric acid and perchloric acid [29]. Total potassium was determined after digestion with hydrofluoric acid and perchloric acid, followed by emission spectrometry analysis [30]. Particle size distribution was determined using sodium hexametaphosphate and sodium carbonate as deflocculating agents to separate different-sized particles: sand (2.0–0.05 mm), silt (0.05–0.002 mm) and clay (<0.002 mm) [31]. Examination of all soil samples was performed in duplicate. A part of the remaining soil samples was stored at 4 °C for isolation, while the other portion was kept at −20 °C for DNA extraction.

### 2.2. Analysis of Soil Acid and Alkaline Phosphatase Activity

The potential acid and alkaline phosphatase activities were determined according to [32] using 4-Nitrophenyl phosphate bis salt (PNPP, Sigma, Saint Louis, MO, USA) as a substrate. Briefly, phosphatase activity was measured in triplicate by incubating 1 g of soil with PNPP in a modified universal buffer (pH 4 for acid, pH 11 for alkaline phosphatase assay) at 37 °C. After 1 h, samples were filtered through filter paper (8–13 µm particle retention) and absorption was measured using a spectrophotometer at 405 nm.

### 2.3. Soil DNA Extraction and Quantification of phoD Gene Abundance

Genomic soil DNA (gDNA) was extracted from 0.25 g of fresh soil using NucleoSpin^®^ DNA Stool Kit (Macherey Nagel, Düren, Germany) according to the manufacturer’s protocol and stored at −20 °C. Concentration and quality of total DNA was determined by an Implen Nanophotometer (Los Angeles, CA, USA).

The *phoD* gene abundance was determined by quantitative PCR (qPCR) using a Real-Time PCR System. For absolute quantification, a plasmid standard was created by cloning amplified *phoD* fragment (primers phoD-F733: 5′-TGGGAYGATCAYGARGT-3′ and phoD-R1083: 5′-CTGSGCSAKSACRTTCCA-3′) [10] of a *Bacillus megaterium* YC4-R4 isolate into the pJET1.2/blunt Cloning Vector with CloneJET PCR Cloning Kit (Thermo Fisher Scientific, Waltham, MA, USA) and competent *Escherichia coli* INVα cells. The plasmid standard was sequenced for verification and plasmid concentration was determined and converted to copy number of DNA molecules per µL. Seven serial ten-fold dilutions were prepared to construct standard curves. The copy number of the *phoD* gene in gDNA samples was determined according to standard curves as per gram of soil.

qPCR was performed on Stratagene Mx3000P qPCR System (Agilent Technologies, Santa Clara, CA, USA). All qPCRs were run in triplicate in 25 µL total volume containing 12.5 µL ABsolute qPCR SYBR Green Mix (Thermo Fisher Scientific, Waltham, MA, USA), 1.75 µL of each primer (phoD-F733 and phoD-R1083 at 10 μΜ), 2 µL of 30 µL total DNA from 0.25 g soil and nuclease free sterile water. The qPCR conditions were as follows: 95 °C for 10 min 40 cycles of 95 °C for 15 s, 55 °C for 30 s, 72 °C for 35 s and 72 °C for 8 min. Data was collected during the annealing step. A melting curve analysis was performed to ensure specificity of the reaction: 95 °C for 30 s, 60 °C for 30 s, 80 cycles of 60 °C for 10 s increased by 0.5 °C per cycle.

### 2.4. Isolation of Inorganic and Organic Phosphate Solubilizing Microbes (PSM)

Inorganic phosphate solubilizing bacteria (IPSBs) were isolated on Pikovskaya’s agar (PVK) [33] of the following composition: glucose 10 g; tricalcium phosphate (TCP) 5 g; ammonium sulphate 0.5 g; sodium chloride 0.2 g; potassium chloride 0.2 g; magnesium sulphate heptahydrate 0.1 g; ferric sulphate 0.002 g; agar agar 10 g; adjusted with distilled water to 1 L; the pH was adjusted to 7.0. Organic P mineralizing bacteria (OPMB) were screened on yolk medium (YM), consisting of constituents: peptone 10.0 g; NaCl 5.0 g; beef extract 10.0 g; fresh egg yolk one; agar 18.0 g; adjusted with distilled water to 1000 mL [34]. After 7 days of incubation at 30 °C colonies surrounded by clear halo zones were screened as PSMs, purified and further processed to molecular characterization.

### 2.5. Isolation of phoD-Harbouring Bacterial Strains

Isolation of *phoD*-harbouring bacterial strains was performed by colony screening with PCR. Prior to PCR, serial dilutions of the samples were made with sterile water and plated on nutrient agar. After 3 days of growth at 28 °C, PCR was applied to 50 colonies in the case of each soil sample using *phoD* gene-based universal primers. PCRs were performed in 10 uL final volume using DreamTaq DNA Polymerase (Thermo Fisher Scientific, Waltham, MA, USA) according to the manufacturer’s protocol. PCR conditions were as follows: 95 °C for 1 min 35 cycles of 95 °C for 30 s, 55.5 °C for 30 s, 72 °C for 30 s and 72 °C for 1 min.

### 2.6. 16S rRNA Gene Sequencing and Analysis

Study of bacterial phylogeny, 16S rRNA gene sequencing of the isolated bacterial strains was performed using the 16S rRNA gene specific universal primers 27F and 1492R [35].

### 2.7. Next Generation Sequencing (NGS) and Bioinformatics Pipeline

Bacterial communities of soil samples were assessed via high-throughput sequencing of the 16S rRNA gene on Illumina Miseq platform at UD-GenoMed Ltd. (Debrecen, Hungary). 16S rRNA gene was amplified from the extracted gDNA of soil samples using 16S Amplicon PCR Forward (5′-TCGTCGGCAGCGTCAGATGTGTATAAGAGACAGCCTACGGGNGGCWGCAG-3′) and Reverse (5′-GTCTCGTGGGCTCGGAGATGTGTATAAGAGACAGGACTACHVGGGTATCTAATCC-3′) universal primers (Sigma-Aldrich, St. Louis, MO, USA). Twenty-five cycles of PCR amplification were performed using 12.5 ng DNA and the KAPA HiFi Hot Start Ready Mix (KAPA Biosystems, Wilmington, MA, USA; Roche AG, Basel, Switzerland), with denaturation at 95 °C for 30 s, annealing at 55 °C for 30 s and extension at 72 °C for 30 s. Post-amplification quality control was performed on the Agilent Bioanalyzer (Agilent Technologies, Santa Clara, CA, USA). MagSi-NGSREP Plus (Magtivio B.V., Nuth, The Netherlands) magnetic beads were used to purify 16S rDNA amplicons.

To add Illumina index tags to the ends of the amplicons, Nextera XT Index Kit was used (Illumina, San Diego, CA, USA) with 502, 503, 504, and 701, 702, 703, 704, 705, 706 index primers. PCRs were performed by the KAPA HiFi Hot Start Ready Mix (KAPA Biosystems, Wilmington, MA, USA; Roche AG, Basel, Switzerland) with the following parameters: 8 cycles of denaturation at 95 °C for 30 s, annealing at 55 °C for 30 s and extension at 72 °C for 30 s. PCR products were cleaned up by magnetic beads, and were subjected to library quantification using MagSi-NGSPREP Plus (Magtivio B.V., Nuth, The Netherlands). For the library validation 1 µL of the diluted final library was run on a Bioanalyzer DNA 100 chip on the Agilent Bioanalyzer (Agilent Technologies, Santa Clara, CA, USA). Libraries were normalized, pooled and loaded onto the Illumina MiSeq platform for 2 × 250 bp paired-end sequencing.

The Frogs pipeline was used to process 16S rRNA gene paired-end amplicon reads [36]. Briefly, forward and reverse reads were filtered and merged using vsearch [37] (with the parameters: min amplicon size: 44; max amplicon size: 550; mismatch rate: 0.15). Merged sequences were clustered using swarm [38]. Chimera sequences were removed using remove_chimera.py from the Frogs pipeline. Taxonomic assignment was performed using BLAST [39] against SILVA_SSU_r132_March2018 database [40].

### 2.8. Statistical Analysis

Phosphatase enzyme activities and *phoD* gene abundance data were analysed using R Statistical Software 3.3.1 (R Development Core Team, 2011). Differences between mean values were determined by one-way analysis of variance (ANOVA). Significant differences were determined using student’s *t*-test (*p* < 0.05). The correlations between *phoD* gene abundance, phosphatase activities, abundance of taxa and soil properties were based on Pearson’s correlation coefficients and were calculated in Excel. The pheatmap package was used to plot heatmaps of relative abundance by the corresponding z-score. Z-score was calculated with the formula z = (x − µ)/σ, where x is the abundance of the taxonomic profiles in each samples, µ is the mean value of the abundances and σ is the standard deviation of the abundances.

## 3. Results

### 3.1. Soil Bacterial Community

**Figure 1:**
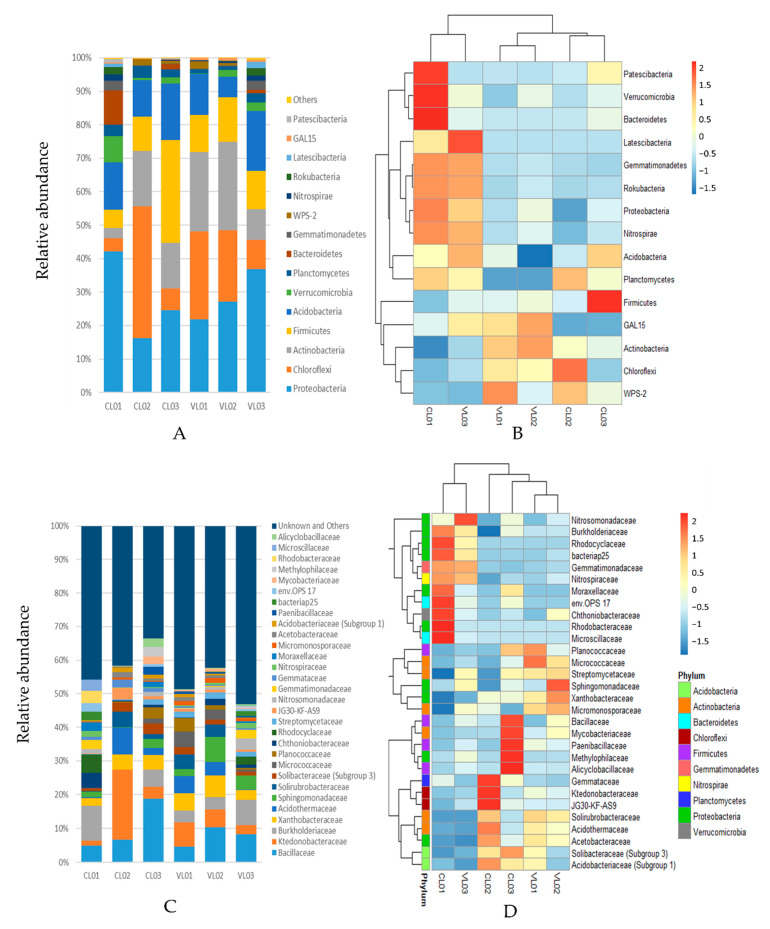
Relative abundance of 16S rRNA gene sequences classified to phylum level: top 15 phyla (**A**), family level: top 30 families (**C**). Heatmap illustrating relative abundance of the 15 most abundant bacterial phyla (**B**) and top 30 families (**D**) in the six soil samples. Relative abundance is indicated by a colour gradient from blue to red with blue representing low abundance and red representing high abundance.

**Figure 2:**
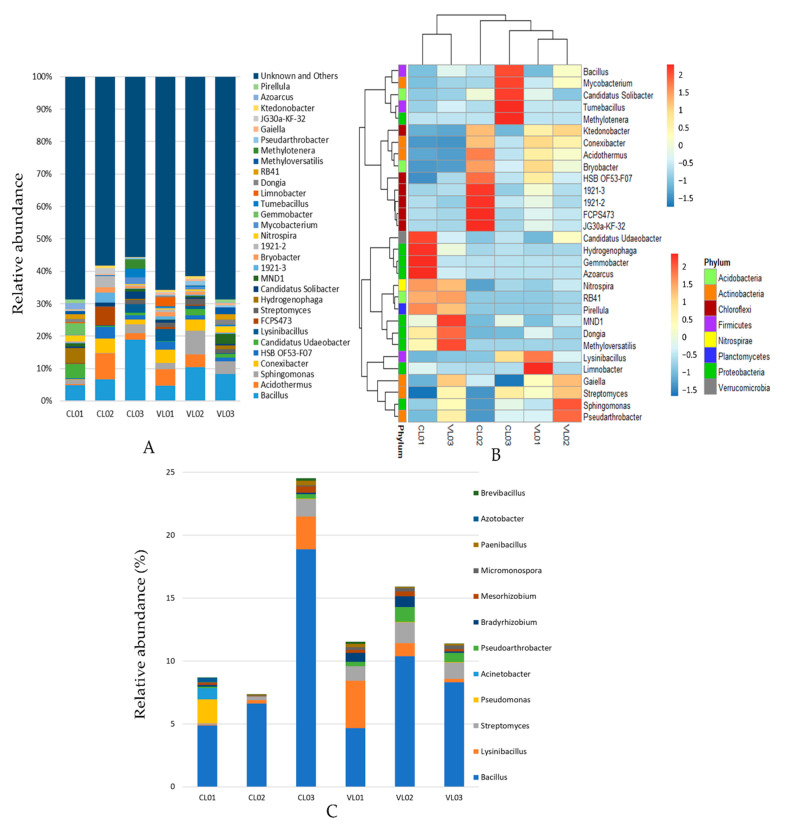
Relative abundance of 16S rRNA gene sequences classified genus level: top 30 genera (**A**) and potential PSB genera (**C**). Potential PSB genera were chosen based on the reports of [7,8]. (**B**) Heatmap illustrating the relative abundance of the 30 most abundant bacterial genera.

We obtained 583,068 sequences from a total of 6 soil samples with a mean of 97,178 reads per sample. The bacterial richness and diversity of the different soil samples were calculated (Table S2). The distribution and abundance of the OTU taxonomic assignments are shown in Figure 1 and Figure 2. In total, 87% of the sequences were assigned to phyla Cholorflexi, Proteobacteria, Actinobacteria, Acidobacteria and Firmicutes. Even though the soil samples shared the same dominant phyla, considerable differences could be observed regarding the abundance of these taxa (Figure 1a,b). Proteobacteria was predominant in soils CL01, VL02 and VL03, Chloroflexi dominated CL02 and VL01, while Frimicutes was predominant in CL03. However, *Bacillus* belonging to phylum Firmicutes was the most abundant known genus in the Firmicutes-dominated CL03 and in the Proteobacteria-dominated samples (CL01, VL02 and VL03) (Figure 1a,b and Figure 2a). Chloroflexi was predominant in samples CL02 and VL01 (Figure 1a,b).

The hierarchically-clustered heatmap showed that the relative abundance of several phyla, such as the dominant Proteobacteria as well as Nitrospirae, Acidobacteria, Rokubacteria, Gemmatimonadetes, Latescibacteria, Planctomycetes, considerably increased in the clusters of CL01 and VL03 compared to the other group consisting of the other four soil samples. On the contrary, other dominant phyla, such as Chloroflexi and Actinobacteria, showed a decreased relative abundance in this cluster compared to the other group (Figure 1b). At the family level, *Burkholderiaceae* and *Gemmatiomonadaceae*, as well as the potential nitrogen-fixing *Rhodocyclaceae*, *Nitrospiraceae* and *Nitrosomonadaceae*, were represented more in the first cluster, while other families were highly represented in one of the samples, such as the dominant families *Bacillaceae* (CL03), *Ktedonobacteraceae* (CL02) and *Sphingomonadaceae* (VL02) (Figure 1c,d). Acidophil taxa, such as *Acidothermaceae*, *Acetobacteraceae* and *Acidobacteriaceae* were detected in the most acidic soil sample, CL02 (pH = 3.9), while acidophil *Allocyclaceae* was the most represented in the also acidic sample CL03 (Figure 1d).

Positive correlation was shown between phyla Proteobacteria, Acidobacteria, Planctomycetes and Firmicutes and the total nitrogen content of soils (Table S3). The same phyla, as well as genera *Bacillus* and *Azotobacter,* positively correlated with silt content, while negative correlation was shown between sand content and the abundance of Proteobacteria, Acidobacteria and Planctomycetes. Genus *Streptomyces,* the ninth most abundant genera in the soil samples correlated negatively with pH (Table S3, Figure 2a). *Streptomyces* species can occur at variable pH, and acidic soil may entail the predominance of the acidophilic *Streptomyces* species (with optima from pH 4.5 to 5.5).

Several phyla and genera showed positive correlation with the copy number of the gene *phoD*, namely phyla Bacteroidetes, Verrucomicrobia, Gemmatimonadetes, Rokubacteria, Nitrospirae, Latescibacteria, Patescibacteria, Zixibacteria, Dadabacteria, Entotheonellaeota, FCPU426 and genera Candidatus *Udaeobacter*, *Hydrogenophaga*, *Gemmobacter*, *Nitrospira*, *Azoarcus*, *Pseudomonas*, *Acinetobacter* and *Azotobacter* (Table S3).

### 3.2. Correlation between Soil Characteristics, Alkaline and Acid Phosphatase Activities, and the phoD Gene Copy Number

**Figure 3:**
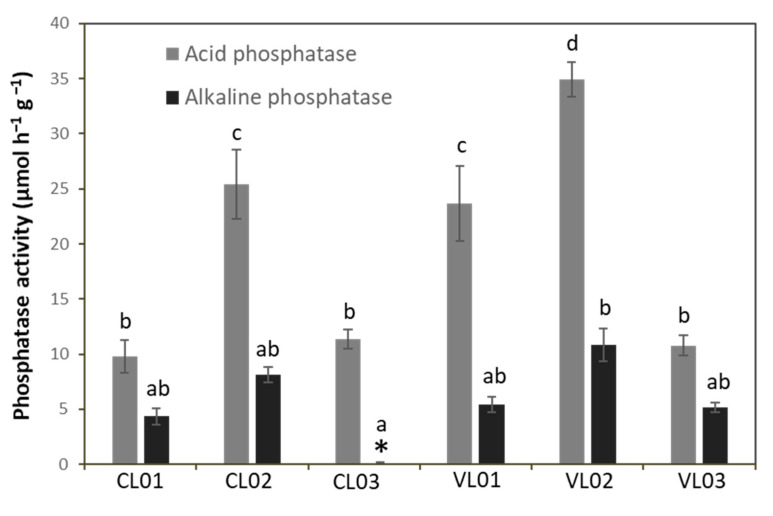
Acid and alkaline phosphatase activity of soil samples. Each value represents the average ± standard deviation (n = 3). Different letters indicate significant differences (*p* < 0.05). Different letters above columns represent significantly different (*p* < 0.05) values. * represents that alkaline phosphatase activity of sample CL03 was under the detection limit.

Sample VL02 showed the highest acid phosphatase activity followed by CL02 and VL01, while that of CL01, CL03 and VL03 was significantly lower than the AcP activity of the other soil samples. No detectable AlP activity could be observed in the case of sample CL03, and we did not find significant difference in the AlP activity of the other samples (Figure 3).

**Figure 4:**
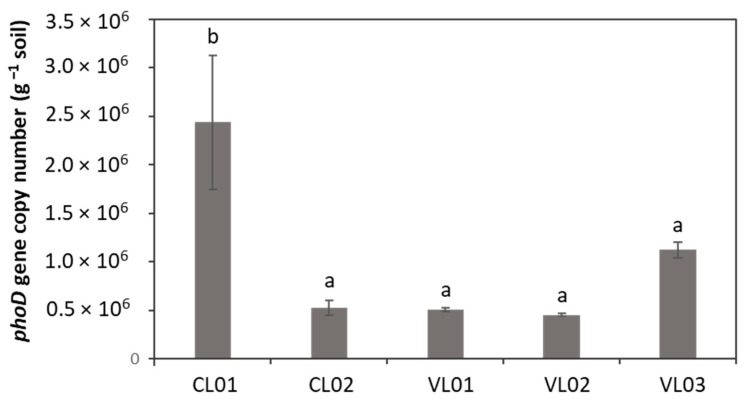
*PhoD* gene abundance. Each value represents the average ± standard deviation (n = 3). Different letters indicate significant differences (*p* < 0.05). No Ct values could be obtained in the case of sample CL03, thus, it was not included in the calculation of *phoD* abundance. Different letters above columns represent significantly different (*p* < 0.05) values.

The abundance of the *phoD* gene was found to be significantly higher in sample CL01 compared to the other soil samples (Figure 4).

**Figure 5:**
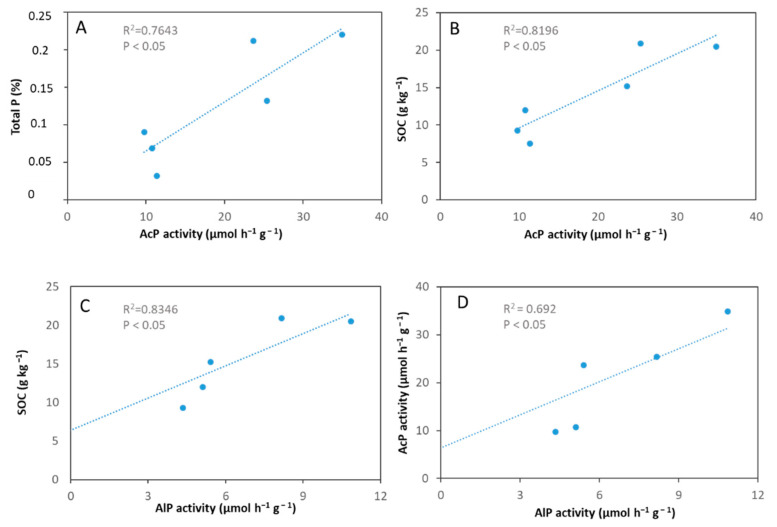
Relationships between the total P content, SOC content, AcP and AlP activity in soils CL01, CL02, CL03, VL01, VL02, VL03. AlP activity of sample CL03 was under detection limit. (**A**) Correlation between total P content and acid phosphatase activity. (**B**) Correlation of SOC content with acid phosphatase activity. (**C**) Correlation between SOC and alkaline phosphatase activity. (**D**) Correlation between acid phosphatase activity and alkaline phosphatase activity.

A significant and positive relationship was found between phosphatase activities and the SOC of soil samples (Figure 5b,c, Table S4, * *p* < 0.05). Besides, AcP significantly and positively correlated with AlP and TP (Figure 5a,d, Table S4, * *p* < 0.05). Surprisingly, no significant correlations were detected between *phoD* gene abundance and alkaline phosphatase activity or other soil properties (Table S4). However, a positive and significant relationship was shown between *phoD* gene abundance and diversity of the soil bacterial community as α-diversity indices, such as Chao1 and Shannon diversity (Table S4) correlated with the copy number of the *phoD* gene.

### 3.3. Isolation of Putative P-Solubilizing and phoD Gene-Harbouring Strains

The 16S rDNA sequencing of isolates showing phosphate solubilizing activity indicated that putative IPSB strains are closely related to *Serratia* (CL02.1, VL02. 1, VL02.2, VL03.3) and *Achromobacter* species (Table S5). Putative OPMB strains were identified as *Stenotrophomonas maltophilia* (CL01.2, CL01.3), *Brevibacillus* sp. (CL02.2, CL03.5, VL01.2, VL01.3), *Bacillus* sp. (CL03.2, CL03.6, CL03.8) and *Lysinibacillus* sp. (CL03.7) strains (Table S5).

*PhoD* gene-harbouring strains showed maximum similarity with *Bacillus* species (CL01.9, CL01.10, CL01.16, CL01.42, CL01.45, VL01.35, VL01.41, VL02.27, VL02.50, VL03.42), *Pseudomonas mendocina* (CL01.16), *Pseudoarthrobacter defluvii* (VL02.34), *Streptomyces lilacinus* (VL02.2) and *Paenibacillus* sp. (VL01.5) strains (Table S6).

## 4. Discussion

### 4.1. Total P, TN, Soil Organic Carbon, Phosphatase Activities and phoD Gene Abundance

Previous studies have suggested that soil acid and alkaline phosphatase activities tend to have a significant relationship with phosphorus, nitrogen and carbon availabilities [9,20,41,42,43]. In the current study, we found a positive correlation between phosphatase activity and SOC (Figure 5, Table S4). This is possibly due to the higher availability of absorbable carbon, which contributes to the development of phosphatase enzyme-producing soil bacteria. Unlike SOC, total phosphorus was found to be a poor predictor of phosphatase activities by Margalef et al. [9], as reservoirs of TP are not a good substrate for phosphatase enzymatic activity. Interestingly, we observed a positive correlation between AcP and TP, which is consistent with the observation of others who showed a positive correlation of TP with AcP [44] and/or AlP activity [20,44]. A potential relationship between bioavailable P and TP can explain this observation, if conversion of P into available forms is enabled in the soil leading to increased phosphatase activities [20,42,44]. High P concentration associated with high organic content may entail higher microbial abundance along with increased production of phosphatases to mineralize organic P into available forms. However, a negative correlation was also shown between available P and AlP activity by Fraser et al. [18,41], suggesting that enzyme production is induced at low available P concentration. Interestingly, Margalef et al., [9] did not show any correlation. Thus, the direct relationship between phosphatase activity and available P could be confusing as it is affected by several chemical and microbiological properties of the soil [42].

Even though TN was found to be a good predictor for acid [9,43] or alkaline [20] phosphatase activity, we could not show a significant relationship (Table S4). Production of phosphatase enzymes—an N-rich molecule—is enabled by high N availability relative to P [9]. The lack of a positive correlation in our study might be caused by the relatively low N availabilities in the soils or by other parameters, such as microbial community composition.

Previous studies found a positive and significant correlation between *phoD* gene abundance and alkaline phosphatase activity [18,20,41,42], suggesting that an abundance of the *phoD* gene might be partially responsible for increased AlP activity. Surprisingly, no significant correlations were detected in our study between *phoD* gene abundance and alkaline phosphatase activity or other soil properties (Table S4). However, AlP assay does not make any difference between extracellular enzymes, and AlP can be encoded by other genes as well (i.e., *phoA*, *phoX*). These genes may have also contributed to the measured AlP activities leading to the lack of correlation with *phoD* abundance in our study. Investigation of the copy number of these genes, and also quantification of the transcript might help to elucidate the response of AlP activity to soil properties. However, quantification of *phoD* transcripts did not reveal any correlation to AlP activity and gene abundance [18]. Even though we did not find a correlation with soil properties and phosphatase activities, a positive and significant relationship was shown between *phoD* abundance and 16s rRNA gene community diversity (Table S3).

In the current study, we found a positive and significant correlation between AcP and AlP activities, which is consistent with the observation of Lemanowicz [43].

### 4.2. Bacterial Community Composition and Correlations with Soil Properties

To the best of our knowledge, this study is the first description of the community structure of bacteria in agricultural soils of Quang Tri province using next generation sequencing. The dominance of Chloroflexi, Proteobacteria, Actinobacteria, Acidobacteria and Firmicutes in soil samples is not unusual. Wei et al. [45] showed the dominance of Proteobacteria, Acidobacteria, Firmicutes and Bacteroidetes in rhizospheric soils of a subtropical forest in China. Proteobacteria and Acidobacteria were also found to be the most abundant phyla in a temperate deciduous broadleaved forest and a tropical mountain rainforest in China [46]. High abundance of Proteobacteria, Actinobacteria, Acidobacteria, Chloroflexi, Gemmatimonadetes, Verrucomicrobia, Bacteroidetes, Planctomycetes, Saccharibacteria and Nitrospirae was found in agricultural and forest soils from China [47]. Proteobacteria and Firmicutes are considered as copiotrophic microbes dominating soils with higher carbon availability with a higher net carbon mineralization rate, whereas Acidobacteria and Chloroflexi, as well as Verrumicrobia and Gemmatimonadetes (also present in our samples), are oligotrophic microbes [48].

The total nitrogen content of soil was shown to affect bacterial communities by several studies [49,50,51]. Here, total nitrogen positively correlated with Proteobacteria, Acidobacteria, Planctomycetes and Firmicutes. An increase in the relative abundance of Proteobacteria was shown by Wang et al. [49] upon N addition in a Chinese fir plantation, confirming the positive effect of nitrogen on this phylum. However, the relative abundance of Acidobacteria decreased due to N addition [49]. Nitrogen content can shape bacterial communities directly as an essential element for microbial growth and indirectly via soil acidification. Proteobacteria is a copiotrophic taxa with a fast growth rate, which might explain the positive correlation of the relative abundance of this taxa with TN. However, TN does not necessarily affect the whole microbial biomass significantly. Unlike TN, a significant effect of total soil organic carbon was shown on the microbial biomass from a primary Korean pine–broadleaved mixed forest [50].

Several studies have demonstrated that differently sized soil particle fractions (clay, silt and sand) are associated with different bacterial and fungal communities [52,53]. Here, we confirmed that certain bacterial taxa showed preference for certain physical niches, namely Proteobacteria, Acidobacteria, Planctomycetes, Firmicutes, *Bacillus* and *Azotobacter* for silty soil. However, other studies showed different correlations compared to our work. For instance, Alphaproteobacteria was shown to prefer a sand-sized fraction of soil by Hemkemeyer et al. [53], while we showed a negative correlation between Proteobacteria and sand content. Hemkemeyer et al. also showed that 16S rRNA gene copies decreased with increasing particle size (clay < silt < sand) [53], which might explain our observation that dominant taxa Proteobacteria, Acidobacteria and Planctomycetes negatively correlated with sand content.

Even though certain reports mention Proteobacteria among the dominant *phoD*-harbouring phyla in soil [10,54,55], no positive correlation was found with the *phoD* copy number in our study (Table S3). However, several genera belonging to phylum Proteobacteria showed a positive correlation with the abundance of the *phoD* gene.

Among the nitrification-related taxa detected, *Nitrosomonas* species can be involved in nitrification by the oxidization of ammonia into nitrite, while members of *Nitrospira* oxidizes nitrite into nitrate [56,57]. The pH of samples CL01 and CL03 (pH = 7 and 6.6, respectively) is within the optimal pH range of nitrification and growth of these bacteria, unlike the more acidic pH of the other samples (Table 1). Members of *Rhodocyclaceae* display different modes of living, including plant-associated nitrogen-fixation [58]. For instance, the *Azoarcus* genus described as N fixing bacteria [59], was also shown to occur, with the highest relative abundance in CL01 (Figure 2a,b).

Certain taxa detected in higher relative abundance in sample VL02 than in other soils, was reported to be involved in microbial remediation of xenobiotic compounds, such as *Sphingomonadaceae* and genus *Pseudoarthrobacter* (Figure 1c,d, Figure 2b). For instance, microbial degradation of various contaminants, such as polycyclic aromatic hydrocarbons (PAHs) and 4-chlorophenol, respectively, has been shown [60,61]. Several *Sphingomonas* strains are able to metabolize a wide variety of carbon sources, survive low nutrient concentrations and utilize contaminants as nutrients [62].

### 4.3. Potential PSB Strains

In our study we placed special attention on bacterial taxa potentially involved in P solubilization. It is important to note that the technique we used did not allow us to prove and determine the role of the detected taxa in the P cycle. Metagenomic analysis of the soil samples revealed that *Bacillus*, *Streptomyces*, *Lysinibacillus* and *Pseudomonas* were the most abundant among potential PSB genera reported to date. *Bacillus* was the most or second most abundant genus in the soil samples (Figure 2a). Isolation of *phoD* gene-harbouring *Bacillus* species from most soil samples (Table S6) also confirmed the presence of this ubiquitous genus. *Bacillus* species are Gram-positive firmicutes including several species involved in phosphate solubilization and plant growth promotion (PGPR). For instance, phosphate solubilizing and AlP phosphatase activity of *B. subtilis* was demonstrated [63,64,65] and its *phoD* gene-encoded AlP, the archetype of the PhoD family, was characterized as part of the Pho regulon, induced upon phosphate starvation [66]. Genes potentially involved in phosphate solubilization were revealed in other *Bacillus* species as well, such as in *B. aryabhattai*, *B. megaterium* and *B. niacini* [67,68,69].

*Streptomyces* was the second most abundant potential PSB in three soil samples (Figure 2c). One isolate of *Streptomyces lilacinus* was found among the *phoD* gene-possessing isolates (Table S6). In addition to plant-growth promoting abilities, *Streptomyces* species are often associated with P transformation, which may include phosphate solubilization and P mineralization in soil. Besides, large amounts of alkaline phosphatase enzyme are reported to be secreted by *Streptomyces* species. AlP enzymes were purified from some species, such as from *S. griseus*, *S. hygroscopicus* and *S. hiroshimensis* [70,71,72], although these enzymes are not closely related to the *phoD* of *Bacillus subtilis*. The lack of a significant relationship between AlP activity and the *phoD* copy number in our study (Table S4) might be associated with the presence of various genes coding for alkaline phosphatase enzymes.

Genus *Lysinibacillus* was found to be a dominant potential PSB in soils CL03, VL01 and VL02 (Figure 2c). In addition to this, an organic phosphate mineralizing strain *Lysinibacillus* sp. was isolated from soil CL03 (Table S5). Plant growth promoting potential of *Lysinibacillus* species, such as *L. sphaericus*, was reported based on its phosphate solubilizing, nitrification and nitrogen-fixing ability [73]. Besides, a *Lysinibacillus fusiformis* strain applied with sawdust biochar was shown to have a positive effect on maize plant height and nutrient concentrations, such as P, N and K [74].

Soil CL01 seems to contain members of *Pseudomonas* genus in high abundance based on the metagenome sequencing of 16s rDNA (Figure 2). A *phoD* gene possessing strain showing high similarity with *Pseudomonas mendocina* was isolated from the same soil sample (Table S6). Pho-regulated phosphatases PhoX and PhoD of *Pseudomonas fluorescens* have been identified by Monds et al. [75]. In addition, numerous PSB bacteria were previously identified as members of genus *Pseudomonas* with plant growth promoting ability. For instance, the phosphate solubilizing ability of a *P. aeruginosa* strain was reported by Sharma et al., and was reckoned as a potential phosphate solubilizer and biofertilizer in apple crops [76]. Additionally, endophytic PSB *Pseudomonas fluorescens* isolates were shown to stimulate the growth of *Pisum sativum* L. plants [77].

Even though we have no information about the content of plant protection chemicals, heavy metals and other contaminants in our soil samples, the use of pesticides can negatively affect the activity of plant beneficial microbes, including PSBs. However, a study showed the positive effect of the fungicid fluopyram on the number of pepper rhizosphere phosphate solubilizing bacteria [78]. In addition to the plant growth promoting abilities of several phosphate solubilizing microbes, certain strains of PSBs were also shown to immobilize heavy metals in soil, such as *Acinetobacter pittii* gp-1, which can promote the immobilization of lead (Pb) [17].

Phosphate solubilizing microorganisms are essential drivers of P cycling in soil ecosystems. PSBs have the potential to alleviate P scarcity in soil, and therefore enhance crop yields. Determination of the plant growth promoting ability of PSB isolates may enable their use as biofertilizers in the future. Besides, evaluation of their multiple P source utilization capacity may clarify their role in the phosphorus cycle. Alkaline phosphatases and acid phosphatases are among those enzymes that can contribute to the release of available phosphorus forms leading to improved soil fertility. The P cycling-related *phoD* gene coding for the alkaline phosphatase facilitates the adaptation of soil bacteria to the fluctuations of available P concentration in soil. The use of microbes with P sources utilizing abilities, including the production of alkaline phosphatases, might be a new strategy to improve soil quality in a sustainable way.

## 5. Conclusions

Metagenome sequencing results verified that 87% of the total sequences were assigned to phyla Cholorflexi, Proteobacteria, Actinobacteria, Acidobacteria and Firmicutes. We detected several bacterial families and genera potentially involved in the P and N cycle, plant growth promotion or soil remediation. Our study showed a positive relationship between phosphatase activities and SOC as well as between AcP and TP in agricultural soils from Vietnam. A positive relationship was shown between certain phyla and the total nitrogen content of soils. Several phyla and genera showed a positive correlation with the copy number of the gene *phoD*.

Soil microbial community structure and diversity are important indicators of soil health. Our results provide new insights into the microbial diversity and microbial phosphate solubilization in agricultural soils of Vietnam, which might help to promote sustainable agricultural practices in the future.

## Figures and Tables

**Table 1:** Soil properties.

Samples	CL01	CL02	CL03	VL01	VL02	VL03
pH_KCl_	7.00 ± 0.022	3.90 ± 0.036	4.60 ± 0.033	4.00 ± 0.031	5.70 ± 0.051	6.50 ± 0.032
Particle size distribution (%)						
sand	42.45 ± 0.5	37.72 ± 0.5	74.62 ± 0.3	55.66 ± 0.2	73.95 ± 0.2	63.08 ± 0.3
silt	31.43 ± 0.5	40.57 ± 0.5	19.35 ± 0.4	0.98 ± 0.2	1.49 ± 0.2	2.49 ± 0.3
clay	26.12 ± 0.5	21.71 ± 0.5	6.03 ± 0.3	43.36 ± 0.3	24.57 ± 0.4	34.43 ± 0.5
SOC (g kg^−1^)	9.30 ± 0.115	20.90 ± 0.120	7.50 ± 0.210	15.20 ± 0.223	20.50 ± 0.300	12.00 ± 0.300
TN (%)	0.17 ± 0.010	0.19 ± 0.010	0.083 ± 0.010	0.14 ± 0.021	0.13 ± 0.024	0.10 ± 0.013
TP (% P_2_O_5_)	0.09 ± 0.001	0.132 ± 0.004	0.032 ± 0.002	0.212 ± 0.001	0.22 ± 0.003	0.069 ± 0.003
TK (%)	0.25 ± 0.022	0.22 ± 0.013	0.028 ± 0.010	0.14 ± 0.010	0.15 ± 0.013	0.31 ± 0.020

CL01–CL03: samples collected from Cam Lo town; VL01-VL03: samples collected from Vinh Linh town; SOC: soil organic carbon; TN: total nitrogen; TP: total P; TK: total potassium. Each value represents the average ± standard deviation (*n* = 2).

## Data Availability

The data presented in this study are available on request from the corresponding author.

## Supplementary Materials

The following are available online at https://www.mdpi.com/article/10.3390/microorganisms9091796/s1, Table S1: Sample names and crops grown on sampling sites, Table S2: The bacterial α-diversity indices in the soils samples, Table S3: Pearson’s correlations between soil properties, phosphatase activities, *phoD* gene abundance and the abundance of bacterial phyla, Table S4: Pearson’s correlations between soil properties, phosphatase activities and *phoD* gene abundance, Table S5: Identities of the PSB strains, Table S6: Identities of *phoD*-harbouring isolates.
