# Supplementary material for: Metagenomic Analysis of Bacterial Communities in Agricultural Soils from Vietnam with Special Attention to Phosphate Solubilizing Bacteria
Source: Microorganisms. 2021 Aug 24;9(9):1796. doi: 10.3390/microorganisms9091796 (PMC8472641; doi:10.3390/microorganisms9091796)
Supplement: Supplementary file 1 [file microorganisms-09-01796-s001.zip › microorganisms-1281539-supplementary.pdf]

Supplementary data

Table S1. Sample names and crops grown on sampling sites

Soil sample	Plantation	
	Perennial	Annual
CL01	N/A	Maize, beans and peas, loofa, gardening plants
CL02	Rubber plant, jackfruit, avocado, black pepper, lime, banana	Elephant grass, bean, turmeric, ginger, sweet potatoes, taros
CL03	N/A	Legumes, maize, sweet potato, cassava
VL01	Black pepper, jackfruit, tea tree	Sweet potato, taro, maize, purple yam, ginger, turmeric, lesser galangal, chive
VL02	Black pepper, rubber plant, banana, jackfruit	Sweet potato, taro, maize, purple yam, ginger, turmeric
VL03	Tea tree, banana, jackfruit, orange, lime, dragon fruit	Beans and peas, sweet potato, tapioca, sesame

N/A: not applicable, no perennial crop grown in these sites.

Table S2. The bacterial α-diversity indices in the soil samples

Sample	OTU	Chao1	Shannon	Good's coverage (%)
CL01	1496	1622	6.10	99.84
CL02	1121	1223	5.85	99.97
CL03	809	952	5.47	99.49
VL01	1028	1087	5.77	99.89
VL02	934	1017	5.70	99.26
VL03	898	969	5.92	99.63

Table S3. Pearson's correlations between soil properties, phosphatase activities, *phoD* gene abundance and the abundance of bacterial phyla.

	pH	TP	Corg	TN	TK	sand	silt	clay	ALP	ACP	*phoD* abundance	Chloroflexi
pH	1											
TP	0.29	1										
Corg	0.39	0.76	1									
TN	0.12	0.38	0.52	1								
TK	0.19	0.27	0.34	0.95	1							
sand	0.06	0.02	0.19	0.88	0.88	1						
silt	0.12	0.39	0.02	0.63	0.57	0.71	1					
clay	0.09	0.56	0.27	0.24	0.31	0.27	0.49	1				
ALP	0.00	0.77	0.92	0.51	0.45	0.15	0.13	0.37	1			
ACP	0.43	0.87	0.91	0.32	0.12	0.11	0.23	0.17	0.84	1		
phoD abundance	0.77	0.19	0.32	0.42	0.68	0.54	0.28	0.28	0.01	0.46	1	
Chloroflexi	0.58	0.13	0.60	0.70	0.49	0.67	0.66	0.07	0.35	0.33	0.19	1
Proteobacteria	0.19	0.16	0.03	0.82*	0.88*	0.91*	0.83*	0.00	0.02	0.26	0.71	0.47
Actinobacteria	0.68	0.22	0.62	0.69	0.46	0.66	0.59	0.01	0.34	0.38	0.25	0.99
Acidobacteria	0.22	0.12	0.23	0.84*	0.76	0.92*	0.89*	0.07	0.10	0.07	0.32	0.83
Firmicutes	0.60	0.09	0.37	0.62	0.41	0.66	0.80*	0.27	0.08	0.13	0.18	0.95
Planctomycetes	0.24	0.10	0.35	0.82*	0.72	0.85*	0.89*	0.16	0.21	0.04	0.22	0.89
Bacteroidetes	0.65	0.26	0.46	0.39	0.62	0.47	0.44	0.02	0.20	0.48	0.91*	0.22
Verrucomicrobia	0.59	0.26	0.36	0.51	0.71	0.59	0.57	0.05	0.14	0.44	0.90*	0.06
WPS 2	0.68	0.18	0.60	0.68	0.44	0.66	0.60	0.01	0.31	0.35	0.25	0.99
Gemmatimonadetes	0.70	0.29	0.42	0.42	0.66	0.54	0.44	0.08	0.16	0.52	0.96**	0.17
Rokubacteria	0.75	0.30	0.47	0.34	0.60	0.46	0.37	0.06	0.19	0.54	0.96**	0.27
Nitrospirae	0.72	0.28	0.48	0.34	0.60	0.46	0.36	0.08	0.21	0.53	0.95**	0.28
Latescibacteria	0.82	0.37	0.50	0.24	0.53	0.42	0.28	0.14	0.20	0.62	0.97**	0.31
Patescibacteria	0.60	0.29	0.45	0.41	0.62	0.49	0.50	0.08	0.23	0.48	0.88*	0.17
GAL15	0.16	0.38	0.04	0.56	0.67	0.60	0.03	0.71	0.09	0.07	0.64	0.04
Armatimonadetes	0.50	0.03	0.57	0.62	0.44	0.65	0.63	0.05	0.33	0.25	0.17	0.98
Zixibacteria	0.82	0.37	0.50	0.26	0.54	0.43	0.30	0.13	0.20	0.61	0.97**	0.30
Dadabacteria	0.72	0.29	0.46	0.36	0.61	0.47	0.39	0.05	0.19	0.52	0.95**	0.25
Entotheonellaeota	0.66	0.24	0.44	0.40	0.63	0.48	0.43	0.02	0.18	0.47	0.92**	0.22
BRC1	0.51	0.04	0.57	0.67	0.47	0.64	0.71	0.17	0.33	0.28	0.17	0.99
FCPU426	0.64	0.23	0.43	0.42	0.64	0.49	0.44	0.00	0.17	0.46	0.91*	0.21

	Proteobacteria	Actinobacteria	Acidobacteria	Firmicutes	Planctomycetes	Bacteroidetes	Verrucomicrobia	WPS 2	Gemmatimonadetes	Rokubacteria	Nitrospirae	Latescibacteria	Patescibacteria	GAL15	Armatimonadetes	Zixibacteria	Dadabacteria	Entotheonellaeota	BRC1	FCPU426
pH																				
TP																				
Corg																				
TN																				
TK																				
sand																				
silt																				
clay																				
ALP																				
ACP																				
phoD abundance																				
Chloroflexi																				
Proteobacteria	1																			
Actinobacteria	0.42	1																		
Acidobacteria	0.87	0.79	1																	
Firmicutes	0.53	0.94	0.87	1																
Planctomycetes	0.80	0.84	0.98*	0.91	1															
Bacteroidetes	0.75	0.27	0.34	0.12	0.23	1														
Verrucomicrobia	0.85	0.12	0.49	0.04	0.39	0.98	1													
WPS 2	0.42	1.00	0.80	0.95	0.84	0.28	0.12	1												
Gemmatimonadetes	0.78	0.24	0.38	0.10	0.27	0.98	0.98	0.24	1											
Rokubacteria	0.71	0.33	0.29	0.19	0.18	0.98	0.96	0.33	1.00	1										
Nitrospirae	0.71	0.33	0.29	0.19	0.17	0.99	0.97	0.33	0.99	1.00	1									
Latescibacteria	0.64	0.38	0.23	0.25	0.13	0.92	0.90	0.38	0.97	0.98	0.97	1								
Patescibacteria	0.78	0.23	0.39	0.05	0.28	1.00	0.99	0.22	0.97	0.97	0.98	0.90	1							
GAL15	0.54	0.03	0.29	0.09	0.13	0.58	0.56	0.01	0.59	0.57	0.61	0.52	0.55	1						
Armatimonadetes	0.43	0.95	0.80	0.92	0.87	0.26	0.09	0.96	0.18	0.27	0.29	0.27	0.22	0.11	1					
Zixibacteria	0.66	0.37	0.25	0.24	0.14	0.93	0.91	0.37	0.97	0.98	0.97	1.00	0.91	0.53	0.27	1				
Dadabacteria	0.73	0.31	0.31	0.16	0.20	0.99	0.97	0.31	1.00	1.00	1.00	0.97	0.98	0.58	0.26	0.97	1			
Entotheonellaeota	0.75	0.27	0.34	0.13	0.23	1.00	0.98	0.27	0.99	0.99	0.99	0.93	0.99	0.59	0.25	0.94	0.99	1		
BRC1	0.48	0.95	0.83	0.95	0.91	0.20	0.03	0.96	0.14	0.23	0.25	0.27	0.14	0.13	0.99	0.26	0.22	0.19	1	
FCPU426	0.76	0.26	0.35	0.11	0.24	1.00	0.99	0.26	0.98	0.98	0.99	0.92	1.00	0.60	0.25	0.93	0.99	1.00	0.19	1

* Correlation between soil property and abundance of phylum is significant at the 0.05 level.
** Correlation between soil property and abundance of phylum is significant at the 0.01 level.
SOC: soil organic carbon; TN: total nitrogen; TP: total P; TK: total potassium; AcP: acid phosphatase activity; AlP: alkaline phosphatase activity.

Table S4. Pearson's correlations between soil properties, phosphatase activities and phoD gene abundance

	pH	TP	SOC	TN	TK	AlP	AcP	abundance	Chao	Shannon
pH	1									
TP	-0.29	1								
SOC	-0.39	0.76	1							
TN	-0.12	0,38	0.52	1						
TK	0.19	0.27	0.34	0.95**	1					
AlP	0.00	0.77	0.92*	0.51	0.45	1				
AcP	-0.43	0.87*	0.91*	0.32	0.12	0.84*	1			
abundance	0.77	-0.19	-0.32	0.42	0.68	-0.01	-0.46	1		
Chao	0.39	-0.06	-0.14	0.73	0.87*	0,02	-0.25	0.84*	1	
Shannon	0.58	0.02	0.02	0.62	0.82*	0.26	-0.25	0.90*	0.76	1

* Correlation is significant at the 0.05 level.

** Correlation is significant at the 0.01 level.

TP: total P; SOC: soil organic carbon; TN: total nitrogen; TK: total potassium; AlP: alkaline phosphatase activity; AcP: acid phosphatase activity; abundance: *phoD* gene abundance; Chao: Chao1 Richness estimate for an OTU definition; Shannon: Shannon diversity index.

Table S5. Identities of the PSB strains

Strains	PSB type[a]	Most closely related species	Accession number	% identity[b]
CL02.1	IPSB	*Serratia* sp.	MT472058	100
VL01.1	IPSB	*Achromobacter* sp.	MT472068	100
VL02.1	IPSB	*Serratia* sp.	MT472074	100
VL02.2	IPSB	*Serratia* sp.	MT472075	99.9
VL03.3	IPSB	*Serratia* sp.	MT472080	100
CL01.2	OPMB	*Stenotrophomonas maltophilia*	MT472051	99.88
CL01.3	OPMB	*Stenotrophomonas maltophilia*	MT472052	100
CL02.2	OPMB	*Brevibacillus* sp.	MT472059	99.44
CL03.2	OPMB	*Bacillus* sp.	MT472060	99.78
CL03.5	OPMB	*Brevibacillus* sp.	MT472061	100
CL03.6	OPMB	*Bacillus* sp.	MT472062	99.78
CL03.7	OPMB	*Lysinibacillus* sp.	MT472063	100
CL03.8	OPMB	*Bacillus* sp.	MT472064	100
VL01.2	OPMB	*Brevibacillus parabrevis*	MT472069	99.02
VL01.3	OPMB	*Brevibacillus sp.*	MT472070	99.77

[a] IPSB: inorganic phosphate solubilizing bacteria; OPMB: organic phosphorus mineralizing bacteria
[b] 602-1014 bp used for Blast analysis

Table S6. Identities of *phoD*-harbouring isolates

Strains	Most closely related species	Accession number	% identity[a]
CL01.9	*Bacillus* sp.	MT472053	100
CL01.10	*Bacillus niacini*	MT472054	100
CL01.16	*Pseudomonas mendocina*	MT472055	100
CL01.42	*Bacillus* sp.	MT472056	100
CL01.45	*Bacillus* sp.	MT472057	100
CL03.10	*Bacillus* sp.	MT472065	100
CL03.27	*Bacillus* sp.	MT472066	100
CL03.35	*Bacillus* sp.	MT472067	100
VL01.5	*Paenibacillus* sp.	MT472071	99.9
VL01.35	*Bacillus* sp.	MT472072	100
VL01.41	*Bacillus* sp.	MT472073	100
VL02.2.phod	*Streptomyces lilacinus*	MT472076	100
VL02.27	*Bacillus* sp.	MT472077	100
VL02.34	*Pseudoarthrobacter defluvii*	MT472078	99.82
VL02.50	*Bacillus* sp.	MT472079	100
VL03.42	*Bacillus* sp.	MT472081	100

[a] 844-1025 bp used for Blast analysis

.
